# Shift Work Is Not Associated with High Blood Pressure or Prevalence of Hypertension

**DOI:** 10.1371/journal.pone.0015250

**Published:** 2010-12-14

**Authors:** Carla Sfreddo, Sandra Costa Fuchs, Álvaro Roberto Merlo, Flávio Danni Fuchs

**Affiliations:** 1 Postgraduate Program in Medical Sciences, Universidade Federal do Rio Grande do Sul, Porto Alegre, Rio Grande do Sul, Brazil; 2 Hospital São Vicente de Paulo, Universidade de Passo Fundo, Passo Fundo, Rio Grande do Sul, Brazil; 3 Postgraduate Program in Cardiology, Universidade Federal do Rio Grande do Sul, Porto Alegre, Rio Grande do Sul, Brazil; 4 Postgraduate Program in Epidemiology, Universidade Federal do Rio Grande do Sul, Porto Alegre, Rio Grande do Sul, Brazil; 5 Division of Cardiology, Hospital de Clínicas de Porto Alegre, Universidade Federal do Rio Grande do Sul, Porto Alegre, Rio Grande do Sul, Brazil; University of British Columbia, Canada

## Abstract

**Background:**

Working mostly at night has been suggested to be associated with upset of chronobiological rhythms and high blood pressure, but the evidence from epidemiological studies is weak.

**Methods:**

In a cross-sectional survey, we evaluated the association between shift work and blood pressure, pre-hypertension and hypertension. In total, 493 nurses, nurse technicians and assistants, were selected at random in a large general hospital setting. Hypertension was diagnosed by the mean of four automatic blood pressure readings ≥140/90 mmHg or use of blood pressure lowering agents, and pre-hypertension by systolic blood pressure ≥120–139 or diastolic blood pressure ≥80–89 mmHg. Risk factors for hypertension were evaluated by a standardized questionnaire and anthropometric measurements. The association between the shift of work and blood pressure, pre-hypertension and hypertension was explored using univariate and multivariate analyses that controlled for risk factors for hypertension by covariance analysis and modified Poisson regression.

**Results:**

The mean age of the participants was 34.3±9.4 years and 88.2% were women. Night shift workers were older, more frequently married or divorced, and less educated. The prevalence of hypertension in the whole sample was 16%, and 28% had pre-hypertension. Blood pressure (after adjustment for confounding) was not different in day and night shift workers. The prevalence of hypertension and pre-hypertension by shift work was not different in the univariate analysis and after adjustment for confounding (all risk ratios  = 1.0).

**Conclusion:**

Night shift work did not increase blood pressure and was not associated with hypertension or pre-hypertension in nursing personnel working in a large general hospital.

## Introduction

Working mostly at night has been suggested to be associated with deleterious consequences of general health, as a consequence of disturbance of chronobiological rhythms [1]. Disturbances of the circadian sleep rhythm could prevent the dipping pattern of blood pressure [2], [3], and increase the incidence of hypertension. Studies with short duration have found higher ambulatory blood pressure among shift workers [4]. Shorter periods of sleep, which have been described for shift workers, could also lead to higher blood pressure [5], [6].

Despite the biological plausibility and evidence from short term studies, there is very few evidence that acute changes in blood pressure regulation in shift workers lead to chronic hypertension. Epidemiological studies linking shift work to the risk for hypertension are few, old and of questionable quality. Two recent and better designed studies found opposite effects. In a Japanese cohort study, habitual shift workers of a steel company had higher systolic and diastolic blood pressure than habitual daily workers [7]. In contrast the Finnish Twin Cohort [8], showed no association between the usual period of work and incidence of hypertension.

The aim of the present study was to investigate the association between shift work and the prevalence of hypertension and pre-hypertension in the nursing staff of a general hospital.

## Methods

### Ethical statement

The Review Board of the Hospital São Vicente de Paulo from the University of Passo Fundo approved the investigation, and all participants gave their written consent to participate.

### Study design

Nursing personnel (nurses, nurse technicians and nurse assistants) of a large general hospital (Hospital São Vicente de Paulo), Passo Fundo, Brazil, were selected at random during the periodic medical examination between January 2008 and August 2009. A total of 493 individuals, predominantly women (88.2% vs. 11.8%), aged from 19 to 68 years agreed to participate.

A standardized questionnaire was used to investigate aspects pertaining to shift working and other risk factors for hypertension. Certified investigators interviewed the participants at the place of work, using a manual with instructions for every section of the questionnaire and for blood pressure and anthropometric measurements. Ten percent of the interviews were repeated at random for quality assurance, and the data were entered in duplicate.

### Study variables

Demographic (age, self-reported skin color), socioeconomic (years at school, previous employments), and lifestyle (physical activities, smoking and alcoholic beverages consumption) characteristics were recorded. Individuals were categorized as current smokers or non-smokers (never smokers or ex-smokers). The pattern of alcohol consumption in the previous year was investigated by a type/frequency questionnaire. The risk of alcoholic beverages consumption was defined by a binge drinking pattern, i.e., 5 or more glasses of drinking in one occasion. Skin color was categorized as white or non-white. Years at school were categorized as more or less than 8 years. Sleeping hours corresponded to the hours of sleep during 24 h in a typical week.

Shifts of work were characterized as morning, afternoon, and night and the combination of them. Since just a few worked exclusively afternoon shifts, this category was combined with the morning shift.

Blood pressure was measured four times during the interview, using an automatic device (OMRON CP-705), and the average was used in the analyses. Hypertension was defined by systolic blood pressure ≥140 or diastolic blood pressure ≥90 mmHg, or use of blood pressure-lowering drugs. Pre-hypertension was defined by systolic blood pressure ≥120–139 or diastolic blood pressure ≥80–89 mmHg.

Weight was measured within 100 g intervals and height within 0.1 cm using a scale with a stadiometer (model 31, Indústria Filizola AS, São Paulo, Brazil), with the participants wearing light clothes and without shoes. Body mass index (BMI) was calculated by dividing weight by height squared.

### Sample size calculation and statistical analyses

The sample size calculation was done with the Epi-Info software, version 6.04. Assuming a prevalence of hypertension of 14% in daily workers and an increase to 25% in workers in the night shift, a power of 80% and confidence limits of 95%, the sample size was estimated as 486 to reject the null hypothesis. Proportions were compared by Chi-square test and means by the Student t-Test for independent samples. Multivariate analyses were carried out using co-variance analyses and Modified Poisson regression.

## Results

**Figure 1:**
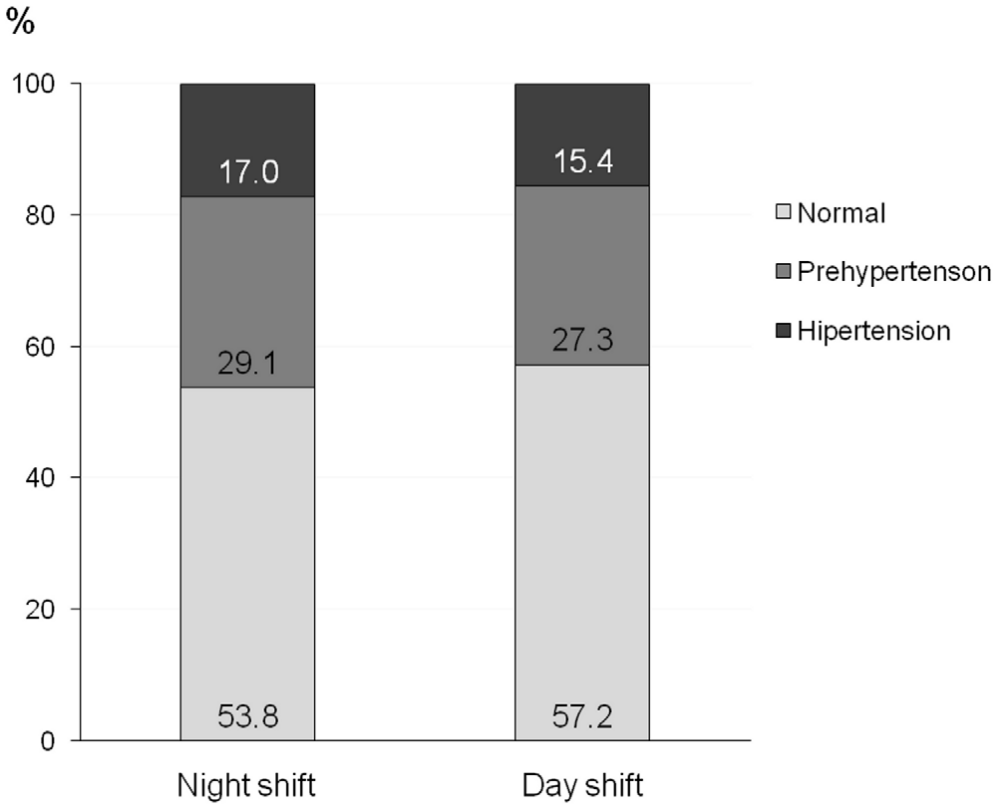
Proportion of the nursing personnel with pre-hypertension and hypertension by shift of work (P = 0.8).

Among 2,140 employees of the hospital, 862 (23.1%) were nurses, nurse technicians or nurse assistants. From these, 493 (57.1%) were selected at random, and none refused to participate. The prevalence of hypertension was 16%, and 28% of the participants had pre-hypertension. Table 1 shows that the participants were relatively young, mostly women, predominantly white, and with an average number of years of education corresponding to high school education. Workers in the night shift were older, less frequently single, and slept more hours per day. Diastolic blood pressure was higher in the night shift workers, but this difference disappeared after adjustment for age, BMI and years at school (P = 0.4). Figure 1 shows that the distribution of hypertension and pre-hypertension by shift work was not different.

**Table 1:** Characteristics of the nursing personnel by shift of work [N (%) or mean ±SD].

	TotalN	Day shift	Night shift	P value*
Gender				0.6
Women	435 (88.2)	276 (88.7)	159 (87.4)	
Men	58 (11.8)	35 (11.3)	23 (12.6)	
Age (years)	34.3±9.4	33.1±9.7	36.4±8.6	<0.001
Skin color				0.9
White	443 (89.9)	279 (89.7)	164 (90.1)	
Non-white	50 (10.1)	32 (10.3)	18 (9.9)	
Marital status				0.006
Single	154 (31.2)	111 (35.7)	43 (23.6)	
Married	270 (56.6)	170 (54.7)	109 (59.9)	
Divorced/widow	60 (12.1)	30 (9.6)	30 (16.5)	
Years at school				0.006
<12	317 (64.3)	186 (58.8)	131 (72.0)	
≥12	176 (35.7)	125 (40.2)	51 (28.0)	
Smokers				0.2
Current	40 (8.1)	16 (5.2)	24 (13.2)	
Never or ex-smokers	452 (91.9)	294 (94.8)	158 (86.8)	
Binge drinking				0.9
No	237 (48.1)	294 (94.5)	172 (94.5)	
Yes	249 (50.5)	17 (5.5)	10 (5.5)	
BMI (kg/m^2^)	25.3±4.7	25.1±4.6	25.8±4.7	0.1
Sleeping (hours)	9,0±3,2	8.6±2.8	9.7±3.7	<0.001
Systolic BP (mmHg)	118.0±14.3	117.8±14.1	118.5±14.6	0.6
Diastolic BP (mmHg)	74.5±10.4	73.8±10.3	75.7±10.6	0.046

*for the comparison between day and night shift.

Table 2 presents the distribution of age, BMI, systolic and diastolic blood pressure, and the classification of blood pressure by the combination of shifts of work. Age and BMI were different by shift of work, without any consistent association with the night shift. Blood pressure and the proportion of participants with hypertension and pre-hypertension were not associated with the shift of work.

**Table 2:** The association between MAEN (*morning, afternoon, evening, night*) working hours with age, BMI and blood pressure.

	Morning or morning and afternoonN = 124	Afternoon and eveningN = 187	NightN = 172	P value
Age (years)	35.0±10.9	31.8±8.5	36.4±8.6	<0.001
BMI (kg/m^2^)	26.1±5.4	24.3±3.9	25.7±4.7	0.001
Systolic BP (mmHg)	118.4±15.7	117.4±12.9	118.5±14.6	0.7
Diastolic BP (mmHg)	73.1±10.9	74.2±9.9	75.7±10.6	0.09
Blood pressure classification			0.8	
Normal	67 (54.0)	111 (59.4)	98 (53.8)	
Pré-hypertension	35 (28.2)	50 (26.7)	53 (29.1)	
Hypertension	22 (17.7)	26 (13.9)	31 (17.0)	

Table 3 presents the adjusted risk ratios for hypertension or pre-hypertension by selected categories and by shift of work characterized as day or night or by the combination of different shifts. Age, male gender and BMI were positively associated with hypertension, while higher number of sleeping hours was associated with lower prevalence of hypertension and pre-hypertension. Shifts of work, either classified as day or night, or by the combination of shifts, were not associated with the prevalence of hypertension or pre-hypertension. The exclusion of sleeping hours from the model did not change the estimates at all. The risk ratios for hypertension alone were similar but had wider confidence limits.

**Table 3:** Risk factors for pre-hypertension or hypertension analyzed by Modified Poisson Regression (Risk ratio and 95% CI).

	RR (95%CI)	P value
Age (years)	1.02 (1.01–1.03)	<0.001
Gender*		
Women	1.0	
Men	2.3 (1.9–2.8)	<0.001
Skin color*		
Non white	1.0	
White	0.9 (0.7–1.2)	0.4
Marital status*		0.08
Single	1.0	
Married	1.4 (0.9–2.1)	
Divorced/widow	1.5 (1.0–2.1)	
Years at school*	0.9 (0.9–1.0)	0.2
Smoking		0.06
Never or ex-smoker	1.0	
Current	1.6 (1.0–2.6)	
Binge drinking*		0.08
No	1.0	
Yes	1.4 (1.0–2.0)	
Sleeping (hours)*	0.96 (0.93–0.99)	0.04
BMI (kg/m2)*		<0.001
<25.0	1.0	
≥25.0	1.9 (1.5–2.4)	
Shift**		0.9
Morning and/or afternoon	1.0	
Afternoon and evening	1.0 (0.8–1.2)	
Night	1.0 (0.8–1.3)	
Shift**		0.7
Day	1.0	
Night	1.0 (0.8–1.3)	

*RR adjusted for age.

**RR adjusted for all above variables.

## Discussion

This cross-sectional survey of a representative sample of the nursing staff of a large general hospital demonstrated that the shift of work was not associated with the magnitude of blood pressure or prevalence of hypertension. The absence of association was confirmed by multivariate analyses and was not influenced by longer periods of total sleep during a typical work week for the nurses that worked the night shift. Traditional risk factors, such as age and BMI, were associated with a higher prevalence of hypertension.

Short term studies with small samples have identified a trend towards higher blood pressure among shift workers [3], [4]. There are few epidemiological surveys with large samples. Two recent cohort studies report opposing findings. Habitual shift workers of a Japanese steel company had higher systolic and diastolic blood pressure than habitual daily workers [7]. However, in the Finnish Twin Cohort, which is a large population-based study with long follow-up, there was no association between the usual period of work and incidence of hypertension [8]. The kind of night shift work and secondary post-hoc analyses of both studies may explain the different findings. Our study was prospectively designed, and had a detailed evaluation of shift of work and blood pressure. The negative findings strengthen the conclusion that work at night, at least in some jobs, such as nursing, is not associated with an increase in blood pressure, hypertension or pre-hypertension. An association between shift work and shorter period of sleeping was expected, but was not confirmed in our survey, since nurses that regularly worked at night reported a longer weekly duration of sleep. Longer periods of sleep could be an intermediate mechanism diminishing the incidence of hypertension in our study, but the absence of an association both in the bivariate and multivariate analyses is against that possible explanation of our findings.

The absence of blood pressure measurements during sleep with an Ambulatory Blood Pressure machine and the lack of an objective evaluation of sleep disorders are possible limitations of our study. The cross-sectional design is not the strongest design to establish the risks of shift work for chronic hypertension, but the absence of any cross-sectional association makes it unlikely that we missed an increase in blood pressure that would develop over time. The fact that the study was limited to nurses makes it impossible to exclude the possibility that hypertension could be linked to shift work in other professions. The prospective planning, adequate power, careful measurement of blood pressure, and evaluation of risk factors for hypertension are strengths of our survey.

In conclusion, night shift work was not associated with an increase of blood pressure, hypertension or pre-hypertension in the nursing staff of a general hospital.
